# Structured P2P Overlay of Mobile Brokers for Realizing Publish/Subscribe Communication in VANET

**DOI:** 10.1155/2014/136365

**Published:** 2014-01-12

**Authors:** Tulika Pandey, Deepak Garg, Manoj Madhava Gore

**Affiliations:** ^1^Deptartment of Computer Science and Information Technology, Sam Higginbotom Institute of Agriculture, Technology & Sciences, Allahabad 211007, India; ^2^Department of Computer Science and Engineering, Thapar University, Patiala 147004, India; ^3^Department of Computer Science and Engineering, Motilal Nehru National Institute of Technology, Allahabad 211004, India

## Abstract

Publish/subscribe communication paradigm provides asynchrony and decoupling, making it an elegant alternative for designing applications in distributed and dynamic environment such as vehicular ad hoc networks (VANETs). In this paradigm, the broker is the most important component that decouples other two components, namely, publisher and subscriber. Previous research efforts have either utilized the deployment of distributed brokers on stationary road side info-stations or have assigned the role of broker to any moving vehicle on ad hoc basis. In one approach, lots of preinstalled infrastructures are needed whereas, in another, the quality of service is not guaranteed due to unpredictable moving and stopping patterns of vehicles. In this paper, we present the architecture of distributed mobile brokers which are dynamically reconfigurable in the form of structured P2P overlay and act as rendezvous points for matching publications and subscriptions. We have taken city buses in urban settings to act as mobile brokers whereas other vehicles are considered to be in role of publishers and subscribers. These mobile brokers also assist in locating a vehicle for successful and timely transfer of notifications. We have performed an extensive simulation study to compare our approach with previously proposed approaches. Simulation results establish the applicability of our approach.

## 1. Introduction

VANET can be defined as a distributed, self-organizing communication network of moving vehicles and stationary road side info-stations. These vehicles are equipped with radio interfaces using which they communicate among themselves or with any existing infrastructure. VANETs can be utilized to disseminate important content which may assist in providing a safe and comfortable driving experience. Efficient and scalable information dissemination remains a major challenge in VANET as communicating nodes may dynamically leave or join the network and availability of any particular node cannot be guaranteed at any given time.

Publish/subscribe communication paradigm [1] is an attractive alternative for designing information dissemination applications in VANET like environment. This paradigm provides decoupling in time, space, and synchronization between information producers, called publishers, and information consumers, called subscribers. Another component called broker acts as a mediator between publishers and subscribers and facilitates decoupled, asynchronous, and anonymous interaction between publishers and subscribers.

Some significant research efforts [2–4] have been made towards publish/subscribe based information dissemination in VANET environment. In these efforts, hybrid VANET architecture is assumed. Road side info-stations (assumed to be connected to the Internet) provide infrastructure services whereas ad hoc vehicle-to-vehicle communication extends the range of these info-stations in areas where infrastructure is not available. In these approaches, either the info-stations are considered to be in the role of brokers or any random vehicle can dynamically become broker at any given time. These efforts contributed significantly towards this body of knowledge and have provided deep insight and understanding of the tradeoffs involved. However, there are some limitations in these approaches which require attention for further scope of improvements.

- For the successful operation of proposed architectures, lots of preinstalled infrastructures are needed. Moreover, these info-stations have to be distributed evenly to cover the entire region under consideration. Otherwise, there may be a situation when information publisher is far away from the interested subscribers and there is no info-station near publisher and/or subscriber. In this situation, getting a suitable broker in time where related publication and subscription can meet is difficult as only vehicle-to-vehicle communication is possible. This may result in loss of important notifications or delayed notifications.
- The strategy of choosing any random vehicle to act as broker may also affect the desired quality of service. As the mobility patterns of the vehicles are unpredictable, there can be a situation where vehicles acting as brokers may take abrupt turn (start moving in opposite direction of subscriber) or may halt, which may lead to loss of (or delayed) notifications.

In this paper, we propose a distributed and reconfigurable mobile broker infrastructure for the notification of publications to interested subscribers. In urban regions like Delhi in India, there are lots of city buses being run by DTC (Delhi Transport Corporation) on various predesignated routes. These routes are also shared by other vehicles. It is observed that at any given time these buses are distributed across the city in such a manner that they cover almost every part of the city. At any route, different buses depart from bus stops every ten minutes. Depending on the time of day which decides the density of traffic, these buses run with certain average speed and on every route a bus can be spotted after a certain distance. In our approach, these buses are assumed to be potentially connected to the Internet and have underlying IP based communication channel among them (e.g., by utilizing infrastructure-based cellular communication, like UMTS). These city buses act as brokers for other vehicles which are in role of publishers or subscribers and can form a distributed hash table (DHT) based P2P overlay for disseminating publications and subscriptions.

The present paper is in the continuation of our on-going studies [5, 6] in this area. The initial idea of utilizing DHT and publish/subscribe together for VANET was coined in [5] and had been extended in [6]. In [6], a study had been performed towards evaluating the performance of system when info-stations are not assumed to be connected to Internet and their DHT structure is maintained by utilizing the ad hoc links formed by moving vehicles between them. It was established that even in this extreme ad hoc environment, without any infrastructure support, the performance of system was acceptable.

The rest of the paper is organized as follows.  Section 2 provides a brief description of background concept such as structured P2P Overlay and DHT.  Section 3 surveys the related works.  Section 4 presents the system model and assumed scenario.  Section 5 provides the protocol details.  Section 6 describes the simulation environment and presents the obtained results.  Section 7 concludes the paper.

## 2. Background Concepts: Structured P2P Overlay

As already discussed before, design of broker component poses a challenge in realizing publish/subscribe for VANET. In such dynamic and large scale settings, a centralized broker is of limited use as it cannot provide desired quality of service and scalability. Essentially, the broker component has to be realized in a distributed manner to achieve scalable application development. In our approach, we form a structured P2P overlay of these broker nodes. P2P systems are essentially application level virtual networks with their own topology and routing procedures. These systems typically utilize the host-to-host virtual connections provided by underlying transport layer to form an abstract overlay network at application layer.

Structured P2P overlays are based on distributed hash table (DHT) and exhibit efficient routing and self-organization capabilities. Implementation of structured P2P networks utilizes consistent hashing mechanisms to assign unique identifiers for participating peers (and the content objects shared by them) from a universal identifier space. There is a predefined relation that maps content identifiers to peer identifiers for identifying responsible peers for storing contents or their references. Thus, searching for any content is a routing process from the requesting peer to the responsible destination peer. For this, each peer maintains some state (in form of a routing table) about other peers to route search requests. Examples of structured P2P systems are Chord [7], Pastry [8], and so forth.

Specifically, we base our design of broker network over Chord DHT [7]. It is one of the earliest, often cited, and very popular structured P2P overlay designs. It provides support for efficiently locating a peer which stores desired contents. It utilizes SHA-1, a consistent hashing mechanism, to assign *m-*bit identifier to each peer and content object. Consistent hashing has several desirable properties essential for the design of Chord DHT. It makes sure that peer identifiers and content identifiers are distributed evenly in the identifier space. Further, taking the value of *m* large enough (*m* = 160), it makes the probability of generating duplicated identifiers for different peers or contents very low. Each peer in Chord calculates its identifier by hashing its IP address whereas content identifiers are generated by hashing the name (name of the file, attribute of the content, etc.) of content object.

In Chord, the *m*-bit identifier space is represented as an *identifier-ring* modulo 2^*m*^. Each peer maintains links to its successor and predecessor peer in the identifier ring. Lookup requests are passed around the ring through successor links. This process terminates when a peer is found which is responsible for the desired identifier getting looked up. In order to improve the routing performance, each peer maintains additional routing information in form of *finger-table*. This finger-table is a routing table of at most *m* entries (for *m*-bit identifier space). The *i*th entry in the finger-table of any peer *n* contains identifier of a peer that succeeds *n* by at least 2^*i*−1^ on the identifier ring, where 1 ≤ *i* ≤ *m* and all operations are modulo 2^*m*^.

As peers leave and join, the routing tables are updated accordingly through cooperative stabilization procedures performed at each peer. This is essential for keeping the structure of overlay intact. Each peer has to periodically exchange messages for refreshing the routing tables to minimize the effect of churn (peer leaving and joining). The maintenance cost is proportional to the churn rate. The higher the churn rate, the shorter the interval of periodic maintenance and the higher the maintenance cost.

## 3. Related Work

Survey of the related literature reveals that very few works have been proposed for information dissemination in VANET which utilize P2P systems and publish/subscribe communication paradigm.

In [9], an approach had been proposed where the city is divided into several segment and each segments forms a separate and interacting Chord DHT based peer-to-peer network of moving vehicles. They have assumed that each vehicle knows its position, direction, and velocity. This information is provided by sophisticated devices such as in-car sensors and navigation systems. Further, the vehicles are equipped with digital maps which assist in triggering the event of crossing the segment border. Vehicle can leave one segmented DHT and join some other while moving. Their approach proves that no knowledge about the network segmentation is required for information dissemination.

Though their approach looks feasible, there are certain limitations. The authors have not provided any supporting simulation results. Moreover, the authors have assumed that within a segment vehicles can directly communicate with each other; that is, all are in the communication range of each other. In such settings, all the vehicles can overhear the messages within a segment and thus the utilization of DHT formation in such small segments needs further investigation.

Some approaches proposed in [2–4] explored the idea of implementing publish/subscribe communication paradigm for information dissemination. Authors of these approaches are using GPS and navigation system-enabled vehicles communicating in a cooperative manner. They have also used info-stations which are connected to the Internet for disseminating information on time and are installed at various positions of the city. They have designed a publish/subscribe middleware where a publisher initiates a notification that includes a point of interest and a persistence area in which the information needs to be disseminated. In these approaches, subscribers subscribe for some content and advertise their location, their future navigation route, and their destination. They take advantage of the navigation system to match the content. After matching, notifications are sent to info-stations through the moving vehicles. All the info-stations are accessible to each other with the help of the Internet. A centralized system attached to a backbone is used to gather all the information and then generates traffic warnings.

PeerTIS had been proposed in [10]. Here all the vehicles are equipped with devices having Internet connections. These vehicles form a structured P2P overlay over the Internet using cellular Internet access to realize scalable information sharing.

In near future, 3G/4G network is going to be used by more number of users which will reduce the available bandwidth per person. Using the same technologies for vehicular communications also may add burden to the already scarce bandwidth. Considering this, the 802.11 working group of IEEE is standardizing 802.11p also known as dedicated short range communication (DSRC) [11] for ad hoc vehicular communication. However, only relying on 802.11p based ad hoc communications for information dissemination may not provide the desired quality of service. This motivated us to make balanced use of both technologies in our approach.

Further, some approaches [12, 13] have been proposed recently which utilize public transport (city buses) for information ferrying between other ordinary vehicles. However, these approaches do not utilize publish/subscribe communication paradigm.

In [12], a two tier architecture is proposed where the upper tier of buses constitutes a mobile backbone for data delivery while the lower tier is composed of ordinary cars and passengers. The authors have argued that city buses in their approach can be considered to behave like wireless mesh routers and establish direct connections among them. Though the presented approach is promising, there are some limitations. How the mobile city buses—with dynamically varying distances among them—can behave like wireless mesh routers and maintain direct connections is not clear. Also, for location services buses use GPS and have digital street map of the city with bus line information.

In [13], an approach is presented where the information dissemination flow is restricted to follow the routes of buses only. The authors have proposed a grid based virtual backbone of city buses. City bus with the longest stay duration is elected as the grid leader which stores the information around the grid. There are many grid leaders in the city and all are connected to each other using ad hoc communication links between each other. All the vehicles in the backbone are assumed to be equipped with a GPS device for location services. The presented approach heavily relies on the durability of ad hoc connections formed between city buses (grid leaders). The approach is feasible only when traffic density is high and distribution of vehicles is uniform across the region. Authors have not evaluated their approach under different traffic conditions, which limits the applicability of the proposed approach.

In our approach, highly sophisticated vehicles equipped with sensors, GPS devices, and digital maps are not required. The information is not sent to any centralized server for generating notifications as proposed in [2–4]. Instead, we are using DHT of the brokers to gather publications generated by vehicles around the city. Further, only the city buses (not all the vehicles) are equipped with cellular Internet interface.

## 4. Assumed Scenario

We have assumed a VANET in urban settings. Each vehicle is equipped with a wireless network interface running IEEE 802.11p with fixed range through which they are able to communicate with each other. The IEEE 802.11p is also known as dedicated short range communication (DSRC) [11] and is an approved amendment to the IEEE 802.11 standard to add wireless access in vehicular environments. The transmission range of vehicles is assumed to be 200 m in our approach. These vehicles are assigned with unique identification numbers. Further, some designated vehicles (city buses) are potentially connected to the Internet and have underlying IP based communication channel among them (e.g., by utilizing infrastructure-based cellular communication, like UMTS). These city buses act as brokers for other vehicles that are in role of publishers or subscribers at any given time. It may be noted that apart from city buses no other normal vehicle is equipped with Internet connectivity.

**Figure 1:**
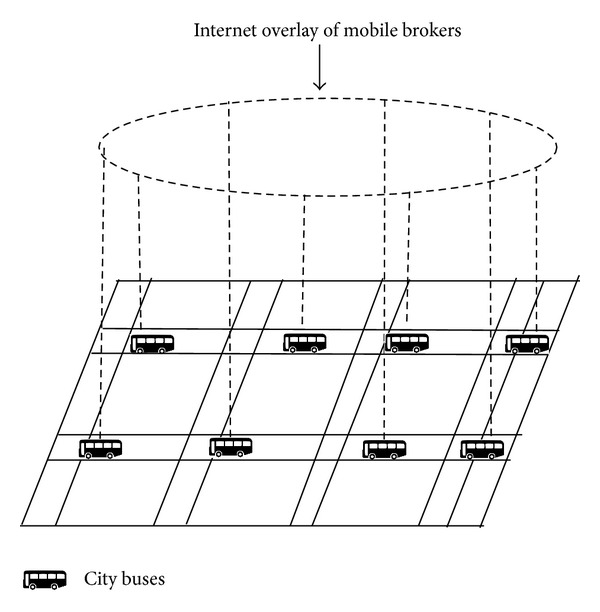
DHT of mobile brokers (city buses).

Figure 1 depicts the assumed scenario where city buses which have fixed timings and generally constant average speeds act as mobile brokers. These mobile brokers act as rendezvous points for publications and subscriptions. These are connected to IP based Internet and form Chord like [7] DHT overlay among them. The IP address associated with each city bus is hashed to find out their logical placement in overlay ring.

In the proposed approach, a vehicle takes the role of publisher when it generates any information. The information can be categorized under a set of predefined topics like “traffic diversions,” “parking space,” “road jams,” and so forth. Similarly, a vehicle may act as a subscriber and express its interest in receiving information related to these topics.

The related publications and subscriptions are routed by underlying Chord DHT of city buses to meet at at-least one broker termed as rendezvous broker. The associated topic names with publications and subscriptions are hashed to get their identifiers. Subsequently, they are routed to and stored at the broker which is the immediate successor of their identifiers. The rendezvous broker performs matching of arriving publications and subscriptions. Matching publications are routed from rendezvous broker towards a broker (using DHT routing mechanism) that is nearest to the current location of subscriber-vehicle. This broker notifies the matching publication to intended subscriber-vehicle using ad hoc vehicle-to-vehicle communication.

## 5. Protocol Details

The proposed protocol design is divided into different procedures. These procedures include the following:

- DHT formation of mobile brokers,
- publication/subscription routing and storage,
- locating the subscriber vehicle,
- notification routing and delivery,
- opportunistic delivery of notification.

The common data structures maintained at each ordinary vehicle are the following:

- *Subscription-Table* and *Publication-Table* for storing its own active subscriptions and publications.
- *Forwarding-Table* for storing subscriptions and publications of other vehicles for forwarding them further.
- *Last-mobile broker* stores the identification of last city bus (with the times-stamp) from which the contact has been established.

Similarly, city buses also maintain *Subscription-Table* and *Publication-Table*. Additionally, the city buses also maintain a *Location-Table* which stores the location information of subscriber vehicles they are responsible for.

The publish primitive in our approach is defined as follows:

*Publish* (*publication-specification*, TTL, *max_hop*).

Here, TTL is the time for which a publication can be considered to be active. The subscribe primitive is defined as

*Subscribe *(*subscription-specification*,* subscriber_IP, *TTL,* max_hop*).

Here subscriber_IP is identification number of the vehicle which has issued the subscription and TTL is the time for which the subscriber should get the matching notification with respect to its subscription.

It may be noted that both publish and subscribe primitives include *max_hop* in their description. This represents the maximum number of hops a publication or a subscription may take when it gets forwarded in ad hoc fashion from vehicle to vehicle. In other words, this also represents that at most in these many hops any mobile broker may be reached. This is required to prevent continuous vehicle-to-vehicle flooding of subscriptions and publications.

### 5.1. DHT Formation of Mobile Brokers

As already mentioned in Section 2, the proposed P2P broker overlay is based on Chord DHT [7]. We assume the presence of a bootstrap server for initialization purpose only. It maintains a list of few city buses recently joined in the system and provides their IP addresses to newly arriving city buses. The city-bus IP addresses can be hashed to a universal identifier space using SHA-1. The uniqueness of city-bus IP addresses and consistent hashing mechanism of SHA-1 ensure that there is less probability of collisions while assigning hashed identifiers.

A city bus attempts to become a part of broker overlay after it starts its journey for a particular route, that is, when it starts from its first stop. Similarly, it detaches itself from the overlay when it reaches its last stop and halts for a stipulated time. The DHT application running on city buses sends hashed value of vehicle identifier to bootstrap server. Bootstrap server keeps record of few mobile brokers which are already in operation and details of these few currently active brokers are provided to a city bus willing to join. This newly arriving city bus forwards the joining request to one of the existing active brokers using its Internet interface. Subsequently, the joining request is routed using DHT routing mechanisms and the new city bus is logically attached to broker overlay at appropriate place. The process of mobile broker joining can be summarized in following steps.

1. Newly arriving mobile broker (*MB*_new_) sends a request to bootstrap server.
2. Bootstrap server replies with the details of an existing mobile broker (*MB*_exist_).
3. *MB*_new_ requests *MB*_exist_ to find the successor of *MB*_new_ in overlay.
4. *MB*_new_ is attached by setting its successor link to point to the successor.
5. *MB*_new_ sets its predecessor link to nil.
6. *MB*_new_ builds its finger-table with the help of its successor.

After the execution of the above steps, newly joined mobile broker *MB*_new_ has its successor link and finger-tables updated. Still, some tasks are required to be performed for the correct operation of overlay structure:

1. setting up the predecessor link of newly joined broker *MB*_new_,
2. updating finger tables, successor links, and predecessor links of existing brokers in overlay, affected by joining of *MB*_new_,
3. transfer of content objects to *MB*_new_ from their successor broker.

These tasks are handled by DHT overlay maintenance procedures. Every node in DHT executes a stabilization procedure periodically. In this procedure, the node sends a request to its successor and asks about the predecessor of its successor. In this manner, two adjacent nodes are able to know if any new node has joined between them. Further, every node executes a fix-finger procedure periodically to update its finger-table. The details of stabilization and fix-finger procedures can be found in [7].

When a mobile broker leaves the network, some procedures have to be followed to make the DHT structure intact. The leave operation is triggered when a city bus is no longer useful in disseminating information. This can happen when it reaches its last stop of route or it gets halted due to any other reason for more than a threshold time limit. Each node in DHT periodically runs a check-predecessor procedure. If there is no response from the predecessor, then the node sets its predecessor as nil. Thereafter, the stabilization procedure can set up the predecessor and successor links of existing nodes accordingly. The details of check-predecessor can be found in [7].

### 5.2. Publication/Subscription Routing and Storage

Generally, the primary goal of vehicles is to transfer their publications or subscriptions to the nearest mobile broker. For this, vehicles utilize vehicle-to-vehicle forwarding mechanism over ad hoc communication links established among them. Every city bus periodically broadcasts a control message in its proximity to inform vehicles of its presence. Once the publications or subscriptions reach any mobile broker they are routed to their respective rendezvous broker through the Internet level DHT formed among them. The following steps are performed by a mobile broker to route publications or subscriptions.

For every publication (*P*) or subscription (*S*) received,

1. extract the value of topic attribute (*t*) from their specification and perform *k* = *hash* (*t*),
2. use finger-table to know the closest successor mobile broker of *k*,
3. send *P* or *S* to the closest successor mobile broker in the DHT,
4. repeat steps 2 and 3 until *P* or *S* reaches the immediate successor of *k*.

For example, suppose that a vehicle issues a publication which has the following specification:

(1) Topic=Road  Jam;  Location=MG  Road;Time  to  Clear=30Minutes;Timestamp=1200 hrs.

As this publication reaches any mobile broker, the value of topic name is hashed and using this hashed, publication identifier, the publication is routed recursively from broker to broker. Finally, it is routed to and stored at the broker whose identifier is the immediate successor of publication identifier.

Now suppose that there is a vehicle that is travelling towards some destination and MG road comes in its route. Before embarking, the vehicle may issue a subscription which has the following specification:

(2) Topic=Road  Jam;Route=Airport−MG  Road−Rajiv  Crossing−Nehru  Place;Timestamp=1155 hrs.

Clearly, the hashed subscription identifier is going to be equal to the hashed publication identifier described earlier. This is due to the consistent hashing mechanism of SHA-1 which creates the same hashed values if the topic names are the same. Consequently, the subscription is also routed to the mobile broker which stores the publication of the same topic name.

This mobile broker acts as a rendezvous point and performs matching operation of publication and subscription looking at the values of other attributes too. In this case, a notification will be generated because MG Road is coming in the route of subscriber vehicle. Further, the timestamp value is also indicating that the subscriber should divert its route for the desired driving comfort and to avoid further traffic congestion on MG Road.

### 5.3. Locating the Subscriber Vehicle

The major challenge in the proposed approach is to locate the subscriber at the time of notification delivery. As the vehicles subscribe for items while moving, there may be a situation that they issue subscription in one region of city and at the time when notification is ready, they are in another region. For successful reception of notification it is essential to locate the subscriber at any given time. In our approach, the location information of subscriber vehicle is maintained and updated in distributed manner over DHT of city buses. The following steps are performed by each subscriber vehicle for updating its location:

1. extract TTL value from subscription specification;
2. repeat until TTL expires OR notification received;
3. **if** (*Last-mobile broker* == broadcasted identifier of city-bus (CB_ID_));
4. **then** (No Location update);
5. **else** (trigger location update);
  - *Last-mobile broker *= broadcasted identifier of city-bus (CB_ID_),
  - *k* = *hash*(*Subscriber* − *VehicleIP*),
  - send *k* and location update request to the city-bus (CB_ID_),
  - city bus uses DHT routing to update location in the *Location-Table* at the immediate successor of *k*.

As already mentioned before, every vehicle maintains a variable *Last-mobile broker* which stores the identification of the last city bus (with timestamp) from which the contact has been established. As a vehicle moves, it comes in proximity of other city buses. This change of location can be known by comparing the stored city-bus ID with the broadcasted city bus ID. If they do not match, then location update is triggered. The value of *Last-mobile broker* is modified and *hash* (*Vehicle* − *IP*) is calculated. This hash value and location update request are forwarded to the city bus in the proximity. Thereafter, the contacted city bus uses DHT routing substrate to update the location in the *Location-Table* at the city bus which is the immediate successor of *hash* (*Vehicle* − *IP*). As a result, certain city buses in DHT are made responsible for maintaining location details of a set of subscriber vehicles. This process gets repeated until the desired notification is received or subscription is invalid due to expiration of TTL.

The location information is maintained only for those vehicles that are in role of subscriber and their subscriptions are active. To know the location of any subscriber-vehicle, the *Location-Table* maintained at the immediate successor of *hash* (*Subscriber* − *Vehicle* *IP*) is looked up in the DHT of city buses. It may be noted that, in our design, subscriber vehicles are not required to be located exactly in a smaller region. Our approach roughly locates the vehicle between two city buses on a specified route.

### 5.4. Notification Routing and Delivery

As already discussed, publications and subscriptions related to the same topics are routed and stored at the same mobile broker. Further, other attributes are utilized for their fine grained matching. Each arriving publication and subscription are matched with the subscriptions and publications already stored in *Subscription-Table* and *Publication-Table*, respectively. In these tables, subscriptions and publications are listed topic-wise.

As publishers and subscribers are purely decoupled, publications and subscriptions can be generated at any time and in any order. Successful notifications strongly depend on ordering of the occurrence of publications and subscriptions, the time instant they reach broker, and their lifespan. In our approach, both publications and subscriptions have definite time spans. These time spans are provided as TTL values in descriptions of publications and subscriptions. Even if the subscription arrives at the rendezvous broker after the publication, subscriber can be notified if subscription lifespan and publication lifespan intersect with each other. The following steps are performed to deliver the notification to subscriber vehicle.

1. Extract *Subscriber_IP* from subscription specification and perform *k* = *hash* (*Subscriber*_*IP*_).
2. Examine the* Location-Table* at the mobile broker that is immediate successor of *k*.
3. Retrieve the location information (ID of mobile broker) of subscriber from *Location-Table.*
4. Use DHT to route the notification to mobile broker found in *Location-Table*.
5. Use ad hoc vehicle-to-vehicle routing to forward notification from mobile broker to subscriber-vehicle.

To forward a notification, a query is sent via DHT lookup procedure to the mobile broker which currently maintains the location details of a given vehicle. This mobile broker gives the identifier of the last-mobile broker contacted by the subscriber-vehicle. The notification is forwarded to that last-mobile broker using underlying DHT routing mechanism. This last-mobile broker forwards the notification utilizing ad hoc routing between other vehicles towards the target subscriber-vehicle.

### 5.5. Opportunistic Delivery of Notification

As discussed earlier, vehicles which need to publish or subscribe forward their publications or subscriptions to their neighbors in 1-hop communication range. These neighbors further forward all the received publications and subscriptions to their 1-hop neighbors. This process continues till the publications or subscriptions reach a mobile-broker. This hop-by-hop transfer of publications and subscriptions towards mobile brokers enables the other forwarder vehicles to act as opportunistic brokers at any given time.

Each ordinary vehicle maintains *Subscription-Table* and *Publication-Table* for storing its own active subscriptions and publications. Further, these vehicles also maintain *Forwarding-Table* for storing subscriptions and publications of other vehicles to forward them further. After receiving publication, subscription, or notification for forwarding, each vehicle matches them with already stored publications and subscriptions. Every received publication is matched with the subscriptions in *Subscription-Table* and *Forwarding-Table*. If the received publication matches with any subscription of *Subscription-Table*, then it is treated as notification for the vehicle itself. If the received publication matches with any subscription in *Forwarding-Table*, then the notification can be generated by the vehicle and forwarded to the subscriber-vehicle. Similarly, opportunistic notifications can be generated by ordinary vehicles when they receive a notification or subscription for forwarding.

## 6. Simulation Setup and Results

We have simulated our approach using Oversim [14] over OMNET++/INET [15]. We have used Chord module of Oversim for simulating DHT of city buses. To generate realistic vehicle movements we have utilized MOVE (MObility model generator for VEhicular networks) [16]. MOVE is built on top of microtraffic simulator SUMO (Simulation of Urban MObility) [17]. To establish communication between SUMO and OMNET++/INET we have utilized TraCI (Traffic Control Interface) [18].

We have chosen OMNET++ because it provides the implementation of the IEEE 802.11p standard which is a recommended protocol for vehicular environment. The important reason behind choosing MOVE is that it provides a GUI for simulating the movement of city buses. Using this GUI the routes of buses, their departure time, speeds, inter-bus interval, and so forth can be defined easily to simulate mobility pattern of city buses. Further, MOVE provides interfaces for realistic road map generation from real world map databases like TIGER (Topologically Integrated Geographic Encoding and Referencing) database or Google Earth. TraCI provides a TCP based client-server architecture. MOVE/SUMO acts as TraCI server whereas OMNET++/INET acts as TraCI client to exchange commands using TCP connections between them.

We have compared our design with an approach closest to ours presented in [2–4]. Here, two reference schemes are discussed, namely, infrastructure persistence and ad hoc persistence. In infrastructure persistence, notifications are stored on road side info-stations and subscriptions are routed towards them. These info-stations are assumed to be connected to a central server which collects publications and issues notifications. In ad hoc persistence, vehicles collaborate with each other to store publications, and subscriptions are routed towards them. Essentially, publish/subscribe paradigm is implemented over infrastructure of info-stations in one scheme and over ad hoc network of moving vehicles in another. Henceforth, the info-station scheme and the ad hoc scheme are referred to as *Comparison-Scenario-1* and *Comparison-Scenario-2*, respectively.

### 6.1. Simulation Parameters

We have performed simulation taking into reference the South Delhi area. This area has organized four-lane road network. We have utilized the Map Editor provided by MOVE which generates real world map for simulation from Google Earth KML (Keyhole Markup Language) files. Further, we have used Vehicle Movement Editor of MOVE to specify the properties of vehicles like vehicle speed, duration of trip, origin and destination of vehicle, vehicle's departure time, and so forth. The simulation parameters are provided in Table 1.

We have simulated the vehicle densities according to realistic traffic situations. For example, during morning and evening hours, traffic density is more due to large number of office goers. Similarly, at night hours traffic density is low. Further, some areas can be considered as hot spots during overall low traffic density period. It is observed that around movie halls, airports, railway stations, night clubs, and so forth traffic density is much more during off hours too. So the distribution of vehicle in the city is normally not uniform. This means that at some places traffic density is high whereas at other places it is low. This is termed as *skewed-distribution* of vehicles in our approach.

The maximum speed of normal vehicles is 65 km/hour whereas the maximum speed of city buses is 45 km/hour. The stoppage time of city buses at bus stops is three simulated minutes. Number of city buses is 15% of total ordinary vehicles at any given time. These buses run according to their fixed routes. The stoppage time of city buses at bus stops is three simulated minutes. The routes of these buses in simulation are specified according to their actual routes information collected from Delhi Transport Corporation (http://www.delhi.gov.in/).

All the ordinary vehicles and city buses have wireless interfaces running IEEE 802.11p protocol. The transmission range of each vehicle is set as 200 meters. City buses also have wireless Internet interface through which they form Chord DHT among them. The parameters for Chord DHT are provided in Table 1. City buses send heartbeat messages every 5 seconds to broadcast their presence to nearby vehicles.

*Comparison-Scenario-1.* For this scenario, static info-stations are created at major intersections in the simulated area of South Delhi. These are also created using Vehicle Movement Editor of MOVE by setting the maximum speed of vehicle (which acts as info-station) as zero. In addition to the wireless interface through which info-stations interact with other vehicles, these are also connected with a central server using TCP/IP interface. The number of city buses is set to zero in this scenario. Further, the subscription specification in this scenario also includes location of vehicle, future navigation route, and destination.

*Comparison-Scenario-2.* For this scenario, both number of city buses and number of info-stations are set as zero. Other simulation parameters are similar to *Comparison-Scenario-1*.

In our approach and in the other two scenarios, any vehicle randomly can take the role of publisher or subscriber. Generally, in realistic situations publishers are less in number than the subscribers. It is assumed that the maximum number of publishers can be 20% of total vehicles whereas maximum 40% can be in role of subscriber. Publications and subscriptions are generated by vehicles at fixed rate by randomly choosing from a predefined set. This set contains matching publication and subscription specifications. Further, the rate of subscription is set to be higher than rate of publication.

### 6.2. Simulation Results

This subsection presents the simulation results. Results are obtained and compared for our approach, *Comparison-Scenario-1*, and *Comparison-Scenario-2* with respect to the following parameters.

1. Notification delivery ratio: this is the ratio of the number of subscriptions for which the successful notifications are received to the total number of subscriptions issued by the subscribers during a given time interval.
2. Notification delay: this is the time required to deliver a notification successfully to intended subscriber.

Further, these results are collected and compared under different simulation settings such as skewed and uniform distribution of vehicles in the simulated region.

#### 6.2.1. Evaluation of Our Approach against Comparison-Scenario-1

**Figure 2:**
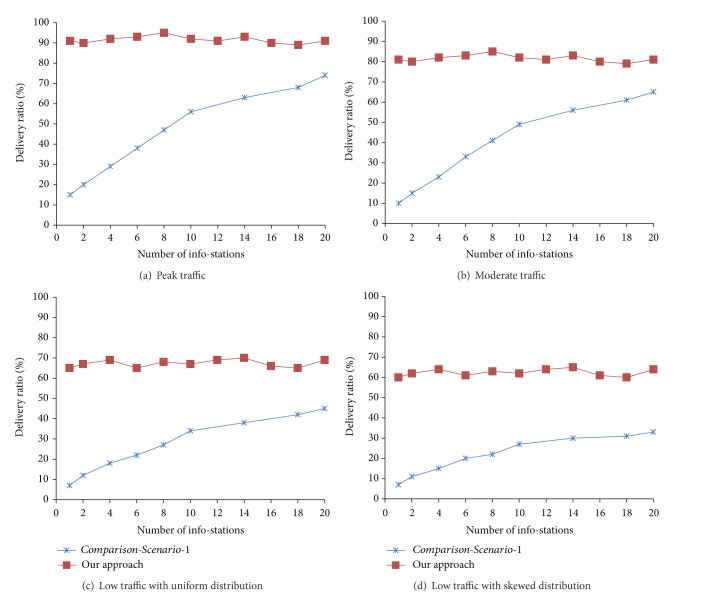
Delivery ratio: our approach versus *Comparison-Scenario-1*.

Figures 2(a)–2(d) depict a set of simulation results where our approach is compared with *Comparison-Scenario-1* with respect to delivery ratio. Here *x*-axis and *y*-axis represent the number of info-stations and delivery ratio, respectively. The delivery ratios shown in results are for successful notifications. As discussed earlier, the subscription and publication both have their associated validity durations. The notification is discarded in route if the validity period is over.

Figure 2(a) shows the results for peak traffic conditions whereas in Figure 2(b) results are presented for moderate traffic conditions. Figures 2(c) and 2(d) show the results for low traffic conditions. In Figure 2(c) vehicles are uniformly distributed across all the roads in simulation area while in Figure 2(d) their population is more skewed towards some hot spots in city such as bars and movie theatres. It may be noted that, in all the traffic conditions, our approach performs better than *Comparison-Scenario-1*.

In peak traffic conditions our approach provides delivery ratio of 89–94% whereas under the same conditions *Comparison-Scenario-1* gives maximum delivery ratio of 74% when 20 info-stations are there in simulated region. This delivery ratio goes down to 47% and 56% when there are 8 and 10 info-stations. Similar patterns may be observed under moderate and low traffic conditions.

Under moderate traffic conditions, our approach gives delivery ratio of 79–85% whereas maximum delivery ratio for *Comparison-Scenario-1* comes as 65% when number of info-stations is 20. Under low traffic conditions with uniform distribution of vehicles, our approach performs between 65% and 72% whereas maximum delivery ratio for *Comparison-Scenario-1* is 45%. Similarly, under low traffic conditions with skewed distribution of vehicles, our approach gives delivery ratio between 60% and 67% compared to maximum delivery ratio of 37% achieved in *Comparison-Scenario-1*.

**Figure 3:**
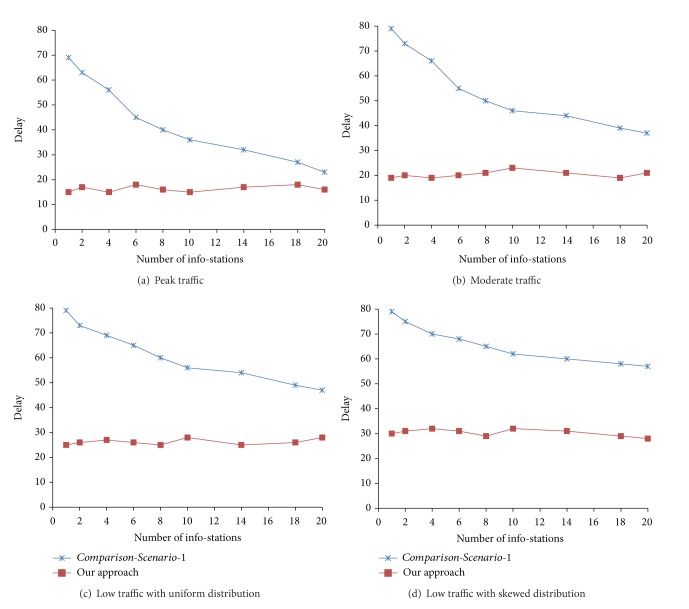
Notification delay: our approach versus *Comparison-Scenario-1*.

Figures 3(a)–3(d) depict a set of simulation results where our approach is compared with *Comparison-Scenario-1* with respect to delay in notification delivery. Here *x*-axis and *y*-axis represent the number of info-stations and notification delay, respectively.

Figure 3(a) shows the results for peak traffic conditions whereas in Figure 3(b) results are presented for moderate traffic conditions. Figures 2(c) and 2(d) show the results for low traffic conditions under uniform and skewed distribution, respectively. It may be noted that, in all the traffic conditions, our approach performs better than *Comparison-Scenario-1*. Further, it may be observed that to reduce the notification delay, *Comparison-Scenario-1* requires more info-stations.

In peak traffic conditions our approach provides notification delay of 15–18 seconds whereas under the same conditions *Comparison-Scenario-1* gives minimum delay of 23 seconds when number of info-stations is 20. This delay is increased to 40 and 36 seconds when there are 8 and 10 info-stations. Under moderate traffic conditions, the notification delay in our approach, 19–23 seconds while minimum delay in *Comparison-Scenario-1* is 37 seconds. Under low traffic conditions (uniform distribution), the notification delay in our approach is between 25 and 28 seconds while for *Comparison-Scenario-1* the minimum delay is 47 seconds. Similarly, under low traffic conditions (skewed distribution), the notification delay in our approach is between 30 and 34 seconds while for *Comparison-Scenario-1* the minimum delay is 57 seconds.

#### 6.2.2. Discussion

It is observed from Figures 2 and 3 that the approach presented in *Comparison-Scenario-1* relies heavily on the number of info-stations. It performs better with more number of info-stations but performance goes down drastically when the number of info-stations is less. This suggests that a lot of preinstalled infrastructures are required to make this approach applicable with desired quality of service.

Further, even when the number of info-stations is substantially large, the performance of *Comparison-Scenario-1* is low compared to our approach. The reason is that vehicles have to move in the transmission range of stationary info-stations to transfer publications or subscriptions. In the case of city buses acting as brokers, both ordinary vehicles and city buses are mobile and transfer delay can be reduced to a large extent if they move towards each other.

The performance of *Comparison-Scenario-1* degrades further at moderate and low traffic densities. This is because when the number of vehicles is less, it results in a longer delay before a vehicle can find another vehicle in its transmission range. Consequently, hop-by-hop ad hoc transfer takes relatively longer time.

#### 6.2.3. Evaluation of Our Approach against Comparison-Scenario-2

**Figure 4:**
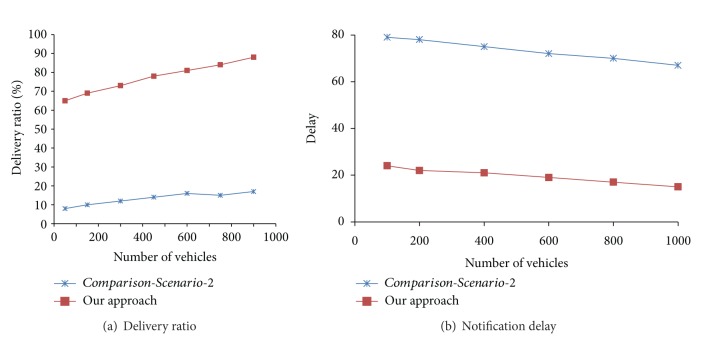
Our approach versus *Comparison-Scenario-2*.

Figures 4(a) and 4(b) depict simulation results where our approach is compared with *Comparison-Scenario-2*. In Figure 4(a), the comparison is shown with respect to delivery ratio. Here, *x*-axis and *y*-axis represent the number of vehicles and delivery ratio, respectively. In Figure 4(b), the comparison is depicted with respect to notification delay. Here, *x*-axis and *y*-axis represent the number of vehicles and delay in notification delivery, respectively.

It may be noted that our approach performs much better than pure ad hoc approach of *Comparison-Scenario-2*. The delivery ratio in our approach is 65–89% compared to 8–17% of *Comparison-Scenario-2*. Similarly, notification delay in our approach is between 15 and 24 seconds compared to 79–67 seconds of *Comparison-Scenario-2*. This is due to the strategy of random assignment of broker role to any vehicle in this scenario. The unpredictable mobility behavior of an ordinary vehicle in role of broker (which cannot be controlled by any external entity) results in notification loss and delay in delivery.

## 7. Conclusion

We have presented our architecture based on publish/subscribe over structured P2P overlay for information dissemination in VANET environment. We have taken city buses to act as mobile brokers whereas other vehicles are considered to be in role of publishers and subscribers. These city buses form DHT overlay among them using their Internet interfaces. We have performed extensive simulation studies and compared our approach with stationary infrastructure based approach and a pure ad hoc approach. We performed realistic simulation analysis by taking map of a region of a metropolitan Indian city. Further, real routes of city buses are simulated to obtain the results. The simulation results suggest that our approach is better compared to infrastructure based approach and pure ad hoc approach.

## Figures and Tables

**Table 1:** Simulation parameters.

(1) Traffic type		
Vehicle density	Time of the day	No. of vehicles
Peak traffic	07:00–10:00 and 17:00–19:00	800 to 1000
Moderate traffic	10:00–17:00 and 19:00–00:00	300 to 500
Low traffic	00:00–7:00	100 to 300

(2) Vehicle properties		

Maximum speed of ordinary vehicle = 65 km/hour Maximum speed of city buses = 45 km/hour City bus pause time at bus stops = 3 minutes No. of city buses = 15% of total vehicles Maximum number of publishers at a time: 20% of total vehicles Maximum number of subscribers at a time: 40% of total vehicles		

(3) Ad hoc communication properties		

Protocol used: 802.11p Transmission range of vehicles: 200 meters Identification broadcast period of city bus: 5 seconds		

(4) DHT communication properties		

Protocol used: chord, underlying protocol: TCP/IP Fix-finger period: Stabilization period:		

(5) For *Comparison-Scenario-1 *		

Central server: one Number of city buses: zero Number of info-stations: variable Transmission range of info-stations: 200 meters Protocol used (for ad hoc communications): 802.11p Protocol used (for infrastructure communications): TCP/IP		

(6) For *Comparison-Scenario-2 *		

Number of city buses: zero Number of info-stations: zero Protocol used (for ad hoc communications): 802.11p Protocol used (for infrastructure communications): TCP/IP		
